# Realtime Remote Programming in Patients Carrying Cardiac Implantable Electronic Devices Requiring Emergent Reprogramming

**DOI:** 10.3389/fcvm.2022.871425

**Published:** 2022-05-16

**Authors:** Shiqiang Xiong, Jin Li, Lin Tong, Jun Hou, Siqi Yang, Lingyao Qi, Xu Chen, Yan Luo, Zhen Zhang, Hanxiong Liu, Lin Cai

**Affiliations:** Department of Cardiology, The Third People's Hospital of Chengdu, Affiliated Hospital of Southwest Jiaotong University, Chengdu, China

**Keywords:** cardiac implantable electronic device, remote programming, emergent programming, telemedicine, COVID-19, follow-up, in-office evaluation

## Abstract

To protect cardiac implantable electronic device (CIED) patients with arrhythmia or possible device malfunction, it is important for health care professionals to provide emergent device evaluation and reprogramming. This case series illustrated the clinical application of realtime remote programming in CIED patients requiring emergent in-person evaluation and reprogramming (ChiCTR2100046883 chictr.org). All remote sessions were performed safely and efficiently by remote electrophysiologists without being in the physical presence of a patient. The implementation of realtime remote programming not only largely reduces the response time to urgent events but also greatly helps to minimize personnel exposure to COVID-19 infection.

## Introduction

Telemedicine, crossing geographic, social, and cultural barriers, has emerged as an important tool for the postimplantation management of patients with cardiac implantable electronic devices (CIEDs). Although remote monitoring (RM) has been classified as I a recommendation for routine use in CIED patients, annual in-office evaluations are also required (1). Limited resources and a seriously imbalanced distribution of follow-up clinics are common hurdles for in-office CIED evaluations (2). The outbreak of the COVID-19 pandemic further induced a drastic reduction in the frequency of in-office evaluations (3). Therefore, the adoption of a prompt response to a patient with a device needing emergent reprogramming remains crucial. From this perspective, we tested an alternative service model using realtime remote programming of CIEDs that would allow for the expeditious and safe testing and programming of dysfunctional cardiac devices without the need for proficient onsite specialists.

## Methods

**Figure 1:**
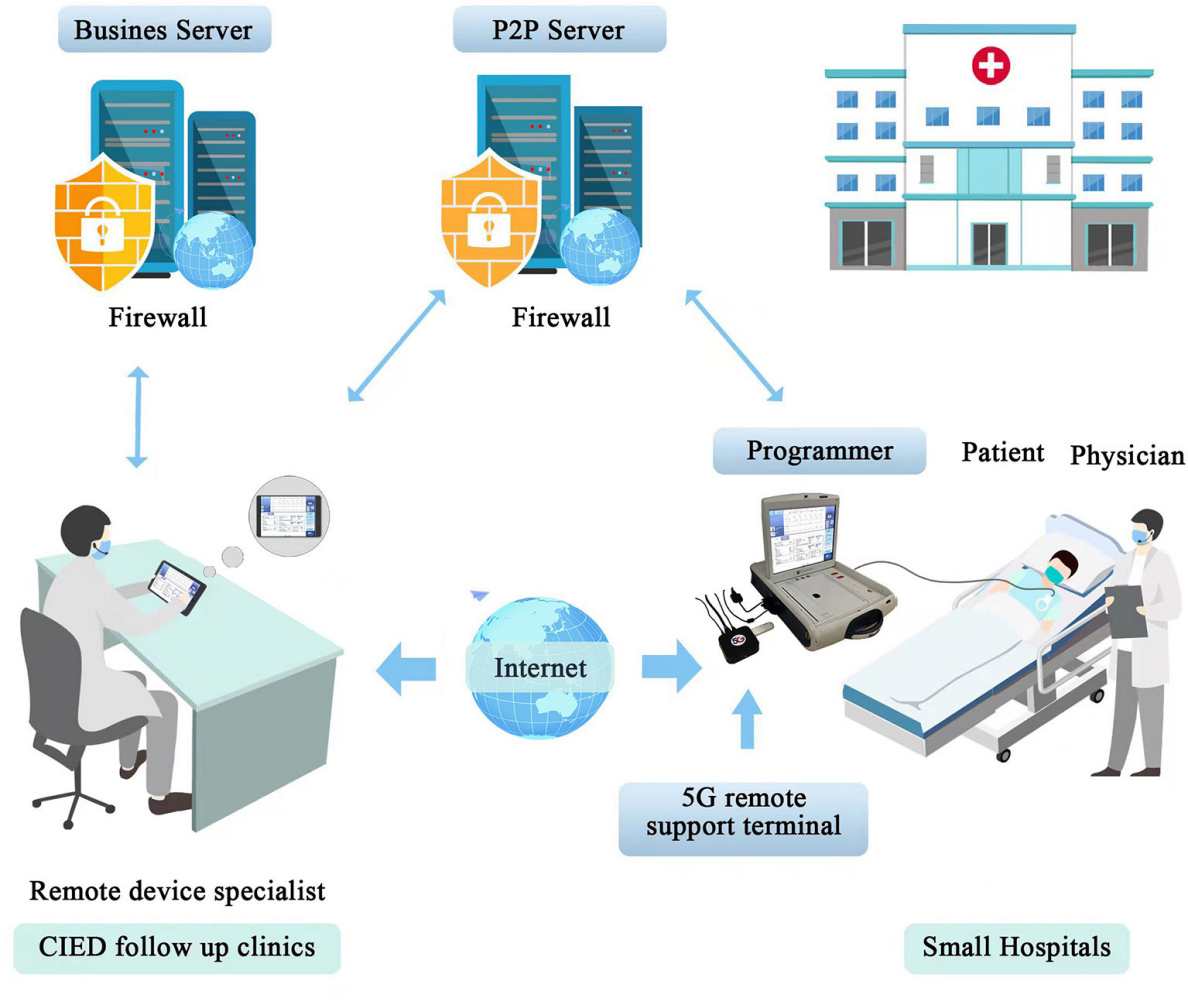
The organization of a realtime remote programming system. The 5G-cloud follow up platform comprises a 5G remote support terminal that is externally connected to the programmer, a PAD installed with a 5G-cloud follow up application, and a remote service system deployed on cloud servers. The P2P Server is used to establish the communication between the 5G remote support terminal and the 5G-cloud follow-up application when the designated account is logged in. Then the 5G remote support terminal is directly connected with the 5G-cloud follow-up application via internet. No network or software is required for the on-site programmer. Remote control of the on-site programmer can be realized by simply connecting to the 5G remote support terminal and using simulated mouse and keyboard information. No direct data interact between the computer and the on-site programmer. The Business Server is used for the second verification to establish remote connection and storing audit logs. This system enables follow up clinics could provide realtime remote programming of CIEDs for small hospitals (primary medical and health care institutions) that lack follow up clinics or device specialists. The application of this system in a hospital includes expeditious remote device programming in different scenarios, such as urgent MR scanning, lead testing during the CIED implantation procedure, and patients in the emergency department. PAD indicates tablet personal computer; CIEDs, cardiac implantable electronic devices; P2P Server, Pointer-to-Pointer Server.

We employed a 5G-cloud follow up platform that allows CIEDs to be evaluated and reprogrammed in realtime from a remote location via an internet connection or a mobile wireless network (Figure 1). The 5G-cloud follow up platform comprised a 5G remote support terminal (China Telecom Corporation Limited Shanghai Branch, Shanghai, China) that was externally connected to the programmer (A Merlin Patient Care System Programmer Model 3650, St. Jude Medical Inc., Saint Paul, Minnesota, USA), a PAD (tablet personal computer) installed with a 5G-cloud follow-up application (China Telecom Corporation Limited Shanghai Branch, Shanghai, China), and a remote service system deployed on cloud servers (China Telecom Cloud, China). Patients were enrolled in the observational trial (ChiCTR2100046883) designed to evaluate the clinical use of cloud follow-up in CIED patients. The study was approved by local ethics committees, and all patients gave informed consent.

The real-time programming session has rigorous security protocols to protect patient safety and cybersecurity. First, the onsite medical staff, after obtaining written informed consent from the CIED patient, began the cloud follow-up session by contacting the remote device specialist via an audio-visual device, and introduced the patient to the remote device specialist. This kind of communication method enabled the remote device specialist to keep in contact with the onsite medical staff and the patient during the whole follow-up session. The onsite medical staff was in charge of precheck of the system, turning on the programmer and applying the programmer wand to the patient's device. Second, a two-step verification was used to log into the 5G-cloud follow-up application on a PAD by the authorized device specialist: Step 1: log into the designated account using a password, and Step 2: use the access password for the second verification to establish remote connection for the designated device. The remote device specialist then had complete control of the programmer functions to evaluate and reprogram the device as appropriate. Third, data transmission was securely encrypted using the RSA/AES 2048-bit asymmetric cryptographic algorithm and sophisticated end-to-end secure communication protocols. Fourth, the servers were deployed in server rooms with protections including multilayer firewalls, customized antivirus scanning, vulnerability scanning and intrusion detection to ensure data security. Fifth, the whole remote operation process was saved via screen recording which allowed users to audit the logs later. Sixth, in case of communication between the on-site programmer and the remote device specialist's PAD is interrupted, the CIED device will revert to the original settings.

## Results

**Case 1** An 83-year-old man with dilated cardiomyopathy and a complete left bundle branch block underwent implantation of a cardiac resynchronization therapy defibrillator (CRTD, Quadra Assura MP^TM^ 3371-40, St. Jude Medical, USA) in May 2019. In September 2021, he was diagnosed with Dukes D stage rectal cancer and accepted expectant treatment. Beginning in October 2021, he suffered from recurrent paroxysms of palpitation, accompanied by occasional shocks. In November 2021, he was admitted to a small hospital due to syncope. At arrival to the hospital, the patient was hemodynamically stable (arterial blood pressure of 100/47 mmHg). A physical examination and an electrocardiogram (baseline rhythm: sinus rhythm/DDD pacing mode, 74 beats/min) did not provide evidence of acute decompensation of heart failure or acute coronary syndrome. Laboratory examinations found the level of hemoglobin was decreased (110 g/L) and the brain natriuretic peptide level was slightly increased (1,786.58 pg/ml). Electrolyte abnormalities and hyperthyroidism were excluded. Because of the lack of qualification to reprogram a CRTD, the local medical staff contacted the device specialist from the author's hospital for emergent technical assistance.

Remote device interrogation demonstrated a total of 64 episodes of ventricular fibrillation (VF), 31 episodes of non-persistent events, and 1 episode of supraventricular tachycardia. The maximum frequency of VF episodes was 45 times within 26 h. Antitachycardia pacing (ATP) terminated 54 of the 64 episodes of VF. Ten episodes of VF were unaffected by ATP and required a shock for termination. The shocks were ineffective in 2 episodes of VF, with successful termination by a subsequent shock.

The device was remotely reprogrammed as follows without the loss of connectivity or programmability: VF shock energy output 2, 30 J to 36 J; value of the VF R-R interval, 12 to 18; VT-2, therapy 2 shock energy output, 15 J to 30 J; VT-2, therapy 3 shock energy output, 30 J to 36 J; left ventricular pulse width 2, 2.5 V to 1.75 V.

**Case 2** A 77-year-old woman with sick sinus syndrome and paroxysmal atrial fibrillation underwent implantation of a single chamber pacemaker (Accent^TM^ SR RF 1,210, St. Jude Medical, USA) in VVIR pacing in July 2019. In December 2021, she presented to the emergency department due to palpitations and general debility. At hospital arrival, the patient was hemodynamically stable (arterial blood pressure 146/84 of mmHg). An electrocardiogram examination detected atrial fibrillation with rapid ventricular rates (130 beats/min). The emergency room physician applied for emergent device evaluation. To minimize personnel exposure to COVID-19 infection, we remotely interrogated and tested the device from the cloud follow up center. The result of remote interrogation found the device was in VVIR pacing mode and the maximum sensor-based rate was 130 beats/min. The ventricular lead parameters were in normal ranges. Before receiving further medication treatment, the atrial fibrillation was autoterminated.

**Case 3** A 69-year-old woman with sick sinus syndrome underwent implantation of a dual-chamber pacemaker (Accent^TM^ DR 2112, St. Jude Medical, USA) in DDD pacing in 2019. In November 2021, she presented to the author's follow-up clinic due to pacemaker syndrome. Device evaluation results showed that her device was in backup VVI mode: the base rate was 67 beats/min, and the ventricular pulse amplitude was 5.0 V. To address this emergent situation, we contacted the manufacturer's representative in Abbott China (Shanghai) for technical support. After obtaining written informed consent from the patient, we began the realtime remote programming session by contacting the remote manufacturer's representative via video call. Once getting the specific password from St. Jude Medical (Sweden), the remote manufacturer's representative successfully reset the device and reprogrammed it to DDD pacing of 60 bpm. We checked with patient's activities, there was no evidence of exposure to strong electro-magnetic field. Since the device have restored successfully, we monitored the patient. At follow-up after realtime reprogramming, the palpitation was completely remitted, and there were no signs of recurrence.

## Discussion

CIED patients who have symptoms suggesting arrhythmia or a possible device malfunction warrant urgent office evaluation. The presented 3 clinical cases varying in scenarios adopted realtime remote programming at the time the device required emergent in-person evaluation and reprogramming. All remote testing and programming sessions were safe and efficient, without any adverse interaction with other aspects of a standard in-office visit. The clinical use of the realtime remote programming of CIEDs provides novel strategies to manage cardiac devices with malfunctions considered urgent or time sensitive.

### The Organization of a Realtime Remote Programming System

The construction of a realtime remote programming system among CIED follow up clinics and small hospitals lacking device specialists may provide substantial benefits for patients needing urgent device reprogramming. In addition, the potential application of this system in a hospital includes expeditious remote device programming in different scenarios, such as urgent MR scanning, lead testing in CIED implantation procedures, and patients in emergency departments. This system enables clinical device specialists to provide rapid and device-specific expertise, without being in the physical presence of a patient.

### The Potential Beneficial Effects of Realtime Remote Programming on CIED Patient Management

Geographic isolation from follow up clinics is a common barrier for in-office CIED evaluations (3). Patients and their caregivers often travel long distances to attend these appointments. During the COVID-19 pandemic, where possible, non-urgent in-office visits should be reasonably avoided (4). Compared with in-office visits alone, RM of CIEDs plus in-office visits resulted in significantly reduced number of unscheduled visits and improved outcomes, without increasing the risk of major adverse events (5, 6). However, as of today, remote programming of CIEDs is not allowed, in view of safety concerns. Realtime remote programming, crossing geographic, social, and cultural barriers, largely reduces the negative effects of geographic barriers and limited resources of follow-up clinics on the postimplantation management of CIEDs. Patients could travel to their local medical institutions and then establish a realtime remote programming session with assigned electrophysiologists who are thousands of miles away. Thus, realtime remote programming may be regarded as an update of routine in-office CIED evaluations and has the potential to improve the management of CIED follow-up.

### The Implementation of Realtime Remote Programming to Minimize Potential Exposure to COVID-19 Infection

In addition to decreasing the response time to urgent events, we implemented realtime remote programming to minimize the potential exposure of medical staff and patients to COVID-19 infection. During the COVID-19 pandemic, the adoption of telemedicine has been rapidly increasing (7). Aiming at the pandemic, RM should be used in most circumstances to reduce the need for non-urgent clinic visits (4). If remote programming is available in the vicinity of a patient's residence or place of work, transregional or long-distance transportation could be avoided. Remote programming of CIEDs enables electrophysiologists to remotely manage CIED patients without the need for a physical presence. This measure contributes to protecting patients and health care teams from COVID-19 exposure. We believe that the integrated application of RM and realtime programming is an ideal organizational model for cardiac device management according to patient profiles, thus minimizing troubleshooting during follow up.

### Communication Protocols to Authenticate and Protect the Connection

The challenges of implementing remote programming of CIEDs are no longer technical (8). The concerns surrounding remote programming are focused on patient safety and cybersecurity issues. The enrolled realtime remote programming system has several layers of protection, including a two-step verification, an asymmetric cryptographic algorithm, sophisticated end-to-end secure communication protocols, and private cloud deployment to protect the cybersecurity of the information and communications. Meanwhile, as an additional safety feature to protect the patient, during each realtime programming session, a physician was always beside the patient to provide assistance, observe the patient, and communicate with the remote electrophysiologist via video/voice call. The onsite medical staff was in charge of turning on the programmer and applying the programmer wand to the patient's device. It is important to remark that the engaged medical staff should know how to troubleshoot and circumvent occasionally arising technical problems.

### Limitations

The present study has some potential limitations. First, as this was a single-center, observational research consisting of only 3 cases, it is insufficient to get the conclusion of safety of the remote programming. Thus, there is a great need for larger studies with rigorous study protocols to confirm this issue. Since the remote programming has not been officially approved for clinical use, clinical researchers of remote programming should strictly abide by the laws and medical ethics. Second, the cloud follow-up system only works with Abbott (St. Jude) devices for the time being, further study extending this service model to other brands of CIED would have greater clinical significance.

## Conclusions

Realtime remote programming is safe and efficient, without any adverse interaction with other aspects of standard in-office visits. The implementation of realtime remote programming not only largely reduces the response time to urgent events, but also has great benefits to minimize potential exposure to COVID-19 infection. The integrated application of RM and realtime programming is an ideal organizational model to ensure optimal CIED management. With the judicious application of this tool, broader applications, along with the further development of new paradigms and protocols are urgently needed.

## Data Availability Statement

The raw data supporting the conclusions of this article will be made available by the authors, without undue reservation.

## Ethics Statement

The studies involving human participants were reviewed and approved by the Ethics Committee of the Third People's Hospital of Chengdu. The patients/participants provided their written informed consent to participate in this study.

## Author Contributions

SX was the major contributor in the collection, analysis and interpretation of data, and drafting of the manuscript. JL, LT, JH, SY, LQ, XC, YL, and ZZ participated in performance of the real time remote programming, and the collection and analysis of data. JL and HL revised the manuscript for important intellectual content. LC designed the study, had full access to all of the data in the study, and finally approved the manuscript submitted. All authors read and approved the final manuscript.

## Funding

This work was supported by the grant from the Science and Technology Department of Sichuan, China (2021YJ0215 and 2020YJ0483), Chengdu High-Level Key Clinical Specialty Construction Project, and the National Natural Science Foundation of China (31600942).

## Conflict of Interest

The authors declare that the research was conducted in the absence of any commercial or financial relationships that could be construed as a potential conflict of interest.

## Publisher's Note

All claims expressed in this article are solely those of the authors and do not necessarily represent those of their affiliated organizations, or those of the publisher, the editors and the reviewers. Any product that may be evaluated in this article, or claim that may be made by its manufacturer, is not guaranteed or endorsed by the publisher.

## Abbreviations

CIEDs: cardiac implantable electronic devices
RM: remote monitoring
VT: ventricular tachycardia
VF: ventricular fibrillation
COVID-19: coronavirus disease 2019.
